# MAEL as a diagnostic marker for the early detection of esophageal squamous cell carcinoma

**DOI:** 10.1186/s13000-021-01098-z

**Published:** 2021-04-26

**Authors:** Mohammad Reza Abbaszadegan, Negin Taghehchian, Azadeh Aarabi, Faride Akbari, Ehsan Saburi, Meysam Moghbeli

**Affiliations:** 1 Medical Genetics Research Center, Mashhad University of Medical Sciences, Mashhad, Iran; 2 Department of Chemistry, Faculty of Science, Ferdowsi University of Mashhad, Mashhad, Iran; 3 Immunology Research Center, Mashhad University of Medical Sciences, Mashhad, Iran; 4 Student Research Committee, Faculty of Medicine, Mashhad University of Medical Sciences, Mashhad, Iran; 5 Department of Medical Genetics and Molecular Medicine, School of Medicine, Mashhad University of Medical Sciences, Mashhad, Iran

**Keywords:** Esophageal cancer, Early detection, Marker, MAEL, cancer testis antigen, Expression, Iran

## Abstract

**Background:**

Esophageal cancer is one of the most common malignancies among Iranians and is categorized as adenocarcinoma and squamous cell carcinoma. Various environmental and genetic factors are involved in this malignancy. Despite the recent advances in therapeutic modalities there is still a noticeable mortality rate among such patients which can be related to the late diagnosis. Regarding high ratio of esophageal squamous cell carcinoma (ESCC) in Iran, therefore it is required to assess molecular biology of ESCC to introduce novel diagnostic markers. In present study we assessed the role of Maelstrom (MAEL) cancer testis gene in biology of ESCC among Iranian patients.

**Methods:**

Forty-five freshly normal and tumor tissues were enrolled to evaluate the levels of MAEL mRNA expression using Real time polymerase chain reaction.

**Results:**

MAEL under and over expressions were observed in 12 (26.7%) and 9 (20%) of patients, respectively. MAEL fold changes were ranged between -4.33 to -1.87 (mean SD: -2.90± 0.24) and 1.92 to 7.72 (mean SD: 3.97± 0.69) in under and over expressed cases, respectively. There was a significant association between stage and MAEL expression in which majority of MAEL over expressed tumors (8/9, 88.9%) were in stage I/II (*p*<0.001). There was also a significant correlation between MAEL expression and depth of invasion in which tumor with T1/2 had higher levels of MAEL expression compared with T3/4 tumors (*p*=0.017). Moreover, there were significant correlations between MAEL expression, tumor size (*p*=0.028), and grade (*p*=0.003) among male patients.

**Conclusions:**

Our data showed that the MAEL was mainly involved in primary stages of tumor progression and it has a declining expression levels toward the advanced stages and higher depth of tumor invasions. Therefore, MAEL can be efficiently introduced as an early detection marker among Iranian ESCC patients.

## Background

Esophageal cancer is the sixth common cause of cancer related deaths among Iranians [1]. Iran is located on the esophageal cancer belt which is spread from China to Iran. Moreover, there is not a homogeneity of esophageal cancer distribution inside Iran, and it has been observed that various ethnics and areas have different age standardized rate (ASR) ranging from less than 3 to about 100 per 100,000 populations [2]. Esophageal cancer is categorized to different subtypes histologically; adenocarcinoma and squamous cell carcinoma (SCC). Various environmental risk factors such as smoking, ethnicity, familial history, and tea temperature are involved in esophageal cancer progression among Iranians [3]. Genetic aberrations have also an important role during ESCC progression among Iranians through deregulation of various cell and molecular processes such as cell cycle, DNA repair, and developmental signaling pathways [4–7]. Despite the recent therapeutic advances, there is still a low 5-years overall survival (about 20 %) among ESCC patients [8, 9]. One of the main reasons of high ratio of mortality in ESCC patients is late diagnosis and lack of severe symptoms in primary stages of tumor progression [6, 10]. Therefore, it is required to determine novel molecular markers to improve the early detection in such patients. Cancer/testis (CT) genes are normally expressed in the germ cells of testis, and aberrantly expressed in various cancers such as esophageal, gastric, and breast tumors[11–13]. Therefore, such limited expression pattern introduces the CT antigens as efficient diagnostic and therapeutic tumor markers [14]. Maelstrom (MAEL) is a cancer/testis-associated gene which is expressed in normal testis and also different tumor tissues such as colorectal and gastric cancers [12, 15, 16]. It is comprised of specific and high mobility domains required for Piwi-mediated silencing [17]. Piwi proteins maintain genetic stability during spermatogenesis by retrotransposon inhibition [18, 19]. Up regulation of some Piwi members have been reported in various cancers [20–23]. The MAEL as a PIWI-interacting protein is associated with regulation of transposable elements expression and DNA damage [24]. Various mechanisms are responsible for the regulation of MAEL expression. Although, the MAEL promoter hyper methylation has been observed in cancer cell lines [15], there was higher MAEL promoter hypo methylation in colorectal cancer (CRC) tissues in comparison with normal margins [25]. It has been observed that the MAEL has a critical function during hepatocellular carcinoma (HCC) progression through AKT/GSK3b/SNAILl signaling. MAEL-transfected cells had increased self-renewal and expression levels of cancer stem cell markers [26]. It stimulates the epithelial–mesenchymal transition (EMT) in colorectal cancer [16]. MAEL promotes the EMT process in bladder urothelial carcinoma via down regulation of metastasis suppressor 1 (MTSS1) which is associated with DNA methyltransferase (DNMT) 3B [27]. It has been also demonstrated that there was a direct association between MAEL expression and ESCC progression [28]. MAEL is involved in reactive oxygen species (ROS) production and stress granule proteins which are associated with DNA damage and apoptosis [29, 30]. Although, MAEL deregulation is associated with liver, colon, gastric, and bladder tumor progression and metastasis [16, 26, 27, 29], it has an inhibitory role in ovarian cancer progression [31]. MAEL has an important role in differentiation of germline stem cells through microRNA-7 (miR-7) inhibition [32]. In present study, we assessed the levels of MAEL mRNA expression in Iranian ESCC patients to determine its probable role during tumor progression and metastasis.

## Methods

### Tissue samples

Forty five tumor and normal fresh tissues were obtained from ESCC patients following the esophagectomy. All of the ESCC cases were confirmed and staged based on tumor-node-metastasis (TNM) system according to the American Joint Committee on Cancer (AJCC) criteria [33]. Inclusion criteria included the presence of at least 80 % of tumor cells in tumor tissues and lack of tumor cells in normal margins. Moreover, all of the normal and tumor tissues should be obtained from the patients who haven’t received any chemo-radio therapeutic treatments before the tumor resection. Tissues were transferred to the RNA later solution (Qiagen, Germany), and stored at -20 ° C prior the mRNA extraction. Informed consent forms which were confirmed by the ethic committee of Mashhad University of Medical Sciences were filled by the patients.

### Real‐time RT-PCR and statistical analysis

CDNA synthesis was performed following the total RNA extraction (Takara, Japan) from the normal and tumor tissues. Comparative real-time PCR (SYBR® Premix Ex Taq™ II kit, TaKaRa) was done in duplicate reactions (Light Cycler, Roche, Germany) with specific primers to assess the levels of MAEL mRNA expression [34]. Glyceraldehyde-3-phosphate dehydrogenase (GAPDH) was applied to normalize data. Thermal profile included an initial denaturing step at 95 °C for 2 min, (95 °C for 30 s and 62 °C for 30 s) 45 cycles, and a final extension step of 72 °C for 30 s. The 2–ΔΔCT algorithm was used to analyze MAEL gene expression. Tumors with more and less than + 2 and − 2 fold changes were considered as over and under expression, respectively. The ± 2 folds interval was also defined as normal expression. SPSS 20.0 statistical package was used for the statistical analyses (SPSS, Chicago, IL). The v2/Fisher exact, independent sample t test, and ANOVA tests were used to assess the correlation between MAEL gene expressions and clinicopathological features (P ≤ 0.05 was considered as significance).

## Results

**Fig. 1:**
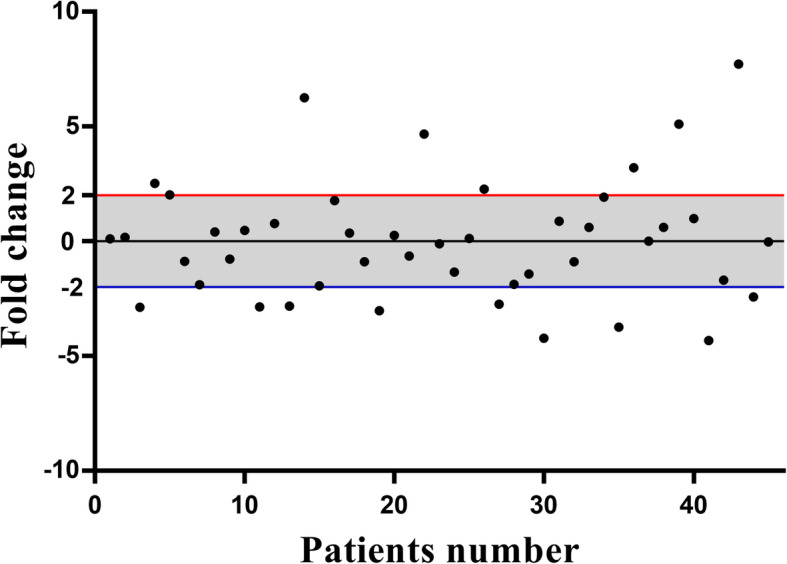
Descriptive analysis of relative MAEL gene expression in ESCC patients. The thresholds for the over and under expressed cases are shown by the red and blue lines, respectively. The grey area mentions to the cases with normal levels of MAEL mRNA expression

In present study, we enrolled 45 ESCC cases (25 males and 20 females) with age range of 30–87 years old (mean ± SD: 61.56 ± 11.38 years old) and tumor sizes were also ranged between 1.5 and 12 cm (mean ± SD: 4.25 ± 1.91 cm). Although, the male patients were older than females (64.8 ± 2.09 VS. 57.5 ± 2.56 years old), the females had bigger tumor sizes compared with males (4.56 ± 0.51 VS. 4.00 ± 0.31 cm). Majority of tumor tissues were moderately differentiated (31/45, 68.9 %), with stage I/II (26/45, 57.8 %), and T3/4 depth of invasion (38/45, 84.4 %). Although, majority of the tumors were T3/4 (84.4 %), less than half (42.2 %) were stage III/IV patients. It was related to the T3 tumors without lymph node metastasis in which 82.6 % of tumors without lymph node involvement were in T3 depth of invasion. There were almost similar ratios of tumors with and without metastatic lymph nodes. Twenty four (53.3 %) and 21 (46.7 %) out of 45 tumors were located in middle and lower esophagus, respectively. All the clinicopathological features are mentioned in Table 1. MAEL under and over expressions were observed in 12 (26.7 %) and 9 (20 %) of patients, respectively. MAEL fold changes were ranged between − 4.33 to -1.87 (mean SD: -2.90 ± 0.24) and 1.92 to 7.72 (mean SD: 3.97 ± 0.69) in under and over expressed cases, respectively (Fig. 1). Probable correlation between levels of MAEL mRNA expression and clinicopathological features of ESCC patients was assessed to clarify the role of MAEL in biology of ESCC. There was not any significant correlation between age and MAEL expression, however we observed that the patients with MAEL under expression were younger than those with over expression (59.25 ± 3.18 VS. 65.89 ± 1.92 years old). Although, there was not any significant correlation between tumor size and levels of MAEL mRNA expression in general population, there was a significant correlation between MAEL expression and tumor size among male patients (*p* = 0.028). In general population, the MAEL under expressed tumors were bigger than the MAEL over expressed tumors (4.94 ± 0.72 VS. 3.48 ± 0.55 cm). There was not any significant correlation between MAEL expression and tumor location in general population. Among males, middle esophagus tumors had noticeably higher levels of MAEL expression compared with tumors which were located in lower (1.38 ± 1.01 VS. -0.22 ± 0.49 fold changes) (*p* = 0.083). In contrast, we have observed that the middle esophagus tumors had lower levels of MAEL expression compared with tumors in lower esophagus in female patients (-1.18 ± 0.58 VS. 0.42 ± 1.12 fold changes). There was not any significant correlation between grade of tumor differentiation and MAEL expression in general population, however we observed a rising trend of MAEL fold changes from well to poorly differentiated tumors. There was a significant correlation between grade and MAEL expression among male patients (*p* = 0.003). Although, there was not any significant correlation between lymph node involvement and MAEL expression, tumors with metastatic lymph nodes had lower levels of MAEL expression compared with those without metastatic lymph nodes (-0.31 ± 0.54 VS. 0.29 ± 0.56 fold changes). There was a significant association between stage and MAEL expression in which majority of MAEL over expressed tumors (8/9, 88.9 %) were in stage I/II (*p* < 0.001). Moreover, the tumors with primary stages (I/II) had higher levels of MAEL expressions compared with advanced stage tumors (III/IV) (0.42 ± 0.50 VS. -0.58 ± 0.61 fold changes). In the case of tumor depth of invasion, there was also a significant correlation between MAEL expression and depth of invasion in which tumor with T1/2 had higher levels of MAEL expression compared with T3/4 tumors (1.54 ± 0.87 VS. -0.29 ± 0.42 fold changes) (p = 0.017). There were not any MAEL under expressed tumors with T1/2 depth of invasion. Moreover, we observed that all of the females had tumors with T3/4 depth of invasion. In the case of sex there was not also any significant correlation between MAEL expression and sex, however it was observed that the males had higher levels of MAEL expression compared with females (0.49 ± 0.53 VS. -0.62 ± 0.55 fold changes).

**Table 1:** Correlation between level of MAEL mRNA expression and clinicopathological features of ESCC patients.

	Total	MAEL over expression	MAEL under expression	MAEL normal expression	*P- Value*
Patients	45	9(20 %)	12(26.7 %)	24(53.3 %)	
Mean age (Years, mean ± SD)	61.56 ± 11.38	65.89 ± 1.92	59.25 ± 3.18	61.08 ± 2.64	0.232
Size (cm, mean ± SD)	4.25 ± 1.91	3.48 ± 0.55	4.94 ± 0.72	4.19 ± 0.33	0.193
Sex					0.252
Male	25(55.6 %)	7(77.8 %)	5(41.7 %)	13(54.2 %)	
Female	20(44.4 %)	2(22.2 %)	7(58.3 %)	11(45.8 %)	
Location					0.245
Lower	21(46.7 %)	3(33.3 %)	4(33.3 %)	14(58.3 %)	
Middle	24(53.3 %)	6(66.7 %)	8(66.7 %)	10(41.7 %)	
Grade					0.749
Well Differentiated	9(20 %)	2(22.2 %)	3(25 %)	4(16.7 %)	
Moderately Differentiated	31(68.9 %)	5(55.6 %)	8(66.7 %)	18(75 %)	
Poorly Differentiated	5(11.1 %)	2(22.2 %)	1(8.3 %)	2(8.3 %)	
Lymph node metastasis					0.564
Yes	22(48.9 %)	3(33.3 %)	6(50 %)	13(54.2 %)	
No	23(51.1 %)	6(66.7 %)	6(50 %)	11(45.8 %)	
Stage					< 0.001
I/II	26(57.8 %)	8(88.9 %)	5(41.7 %)	13(54.2 %)	
III/IV	19(42.2 %)	1(11.1 %)	7(58.3 %)	11(45.8 %)	
Depth of tumor invasion (T)					0.017
T1,2	7(15.6 %)	4(44.4 %)	-	3(12.5 %)	
T3,4	38(84.4 %)	5(55.6 %)	12(100 %)	21(87.5 %)	

## Discussion

**Fig. 2:**
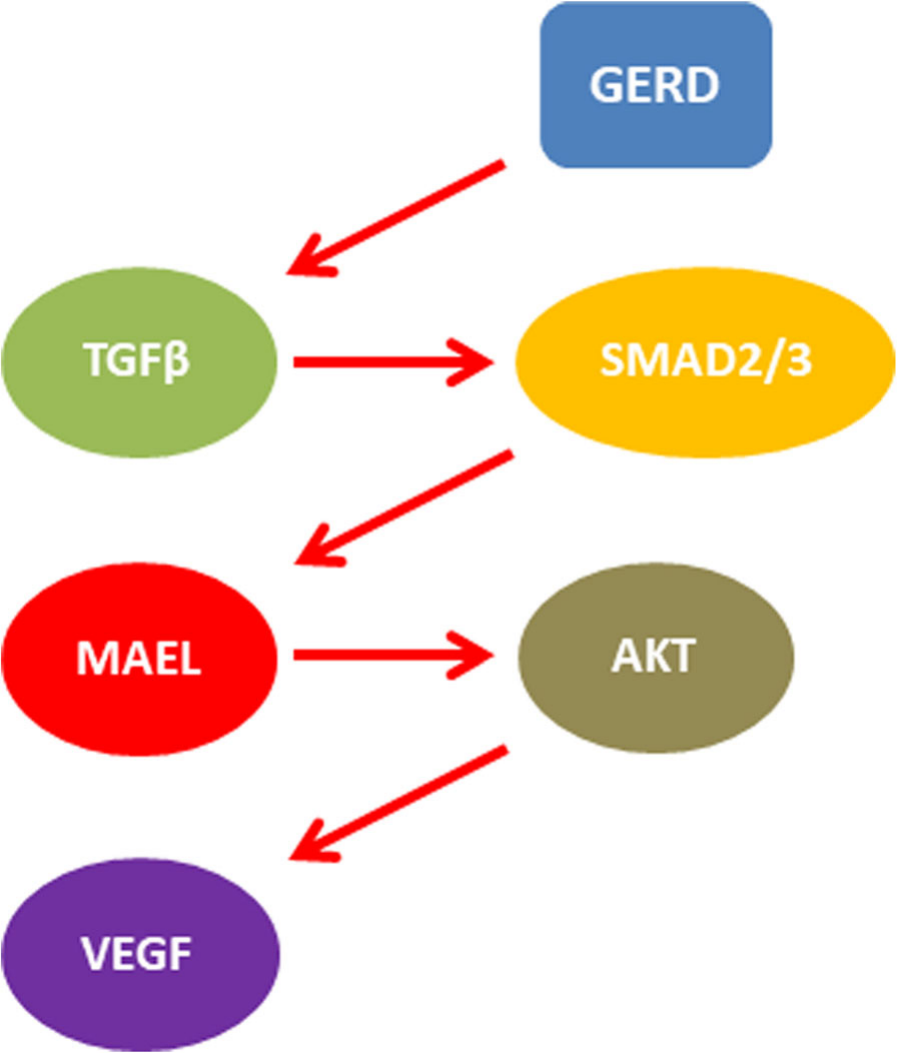
Probable mechanism of higher MAEL levels in lower esophagus among female patients

Esophageal cancer is one of the leading causes of cancer related deaths among Iranian patients which is probably related to the late diagnosis. Therefore, it is required to introduce novel markers for the early detection and targeted therapy. Regarding, the limited expression of CTAs in tumor and normal testis, they have been introduced as the potential therapeutic targets in various cancers [16, 26, 27]. It has been reported that there was MAEL up regulation in tumors compared with normal tissues in ESCC patients. There were also correlations between the levels of MAEL expression and tumor stage, grade, and lymph node metastasis. Moreover, they showed a significant correlation between MAEL and IL8 expressions [28]. IL8 induces tumor metastasis through CXCR1/2 in ESCC [35]. MAEL can be involved in ESCC progression by regulation of AKT1/RelA/IL8 signaling to recruit Myeloid-Derived Suppressor Cells (MDSCs) to tumor sites. Then, the MDSCs up regulate MAEL through TGFb secretion and Smad2/Smad3 phosphorylation in ESCC patients [28]. Another study has been shown an association between MAEL and stress granule proteins in breast and colorectal cancers. Nuage and SGs were assembled due to the germline and somatic stress exposure [36–39] respectively which function as the small RNA-mediated gene silencing locations [40]. The probable role of MAEL in miRNA-mediated gene silencing was highlighted by its presence in the Nuage [24]. MAEL was also associated with chromatin remodeling and transcriptional regulation through interaction with SNF5 and SIN3B [41]. We have recently assessed the expression of MAEL in gastric cancer (GC) patients and showed that there was a correlation between MAEL expression and tumor size. We hypothesized that the AKT activation by MAEL can be resulted in VEGF activation via the mTOR and HIF1a which probably enhanced the angiogenesis and tumor size in GC patients. Moreover, AKT regulated cell proliferation through CCND1. We observed that the primary stage tumors with MAEL over expression had a high aggressive behavior [12]. MAEL has different molecular mechanisms in different tumor types in which it activates the AKT/GSK-3b/SNAIL signaling in HCC [26], inhibits the E-cadherin in CRC [16], inhibits the MTSS1 in bladder cancer [27], and inhibits ILKAP tumor suppressor in GC [42]. EMT is an important cell and molecular process during tumor progression and metastasis [43]. It has been shown that the MAEL suppresses and induces epithelial and mesenchymal markers, respectively. EMT is the primary step of tumor invasion that can be regulated by MAEL in colon cancer. There was a direct correlation between MAEL expression and tumor aggressiveness in colon cancer which can be associated with suppressive role of MAEL in regulation of E-cadherin expression [16]. It has been observed that the high levels of MAEL protein expression was correlated with advanced stages of tumor and poor survival in urothelial carcinoma of the bladder (UCB). MAEL was also involved in EMT process through MTSS1 suppression via DNMT3B in which the DNMT3B recruits the HDAC1 and HDAC2 to the MTSS1 promoter sequence in UCB [27]. MiR-7 is also one of the targets of MAEL which is associated with AKT and EGFR signaling pathways [32, 44]. Genetic stability is associated with DNA repair and chromosomal segregation. Therefore, every aberration in such processes will be resulted in tumorigenesis. MAEL preserves the genetic stability in tumor cells and suppresses Ras-induced senescence [29]. In present study, there were significant inverse correlations between tumor size, grade, and MAEL expression in male ESCC patients. There were also significant inverse correlations between the levels of MAEL mRNA expression, tumor stage, and depth of tumor invasion among all samples in which there were declining trends of MAEL fold changes toward the advanced stages of tumor. Therefore, MAEL has a significant role in primary stages of tumor progression among Iranian patients. There were high levels of MAEL expression in primary tumors with stages of I/II, low depth of invasion (T1/2), and poorly differentiation among Iranian ESCC patients. Interestingly, we observed that the lower esophagus tumors in females had higher levels of MAEL compared with middle esophagus tumors. It seems that the association between the levels of MAEL expression and tumor location in female patients can be related to the higher incidences of Gastroesophageal reflux disease (GERD) among females which prepares an acidic environment in lower esophagus in females. This acidic environment can be associated with TGFb activation that subsequently up regulates the MAEL expression via SMAD2/3 in ESCC [45][28]. Recently we have also reported that the MAEL can be associated with tumor size through up regulation of VEGF in GC patients [12]. Therefore, higher levels of MAEL in lower esophagus and bigger tumor sizes among females can be associated with GERD and acidic condition (Fig. 2).

## Conclusions

In present study we assessed a probable correlation between MAEL expression and ESCC progression among Iranian patients. We showed that the MAEL was mainly activated in primary stages of ESCC progression and there was a declining level of MAEL expression toward the advanced stage tumors. Therefore, MAEL can be introduced as an efficient marker of early detection among Iranian ESCC patients.

## Data Availability

The datasets used and/or analyzed during the current study are available from the corresponding author on reasonable request.

## Abbreviations

ESCC: Esophageal squamous cell carcinoma
MAEL): Maelstrom
ASR: Age standardized rate
SCC: Squamous cell carcinoma
CT: Cancer/testis
TNM: Tumor-node-metastasis
CRC: Colorectal cancer
HCC: Hepatocellular carcinoma
EMT: Epithelial–mesenchymal transition
MTSS1: Metastasis suppressor 1
DNMT: DNA methyltransferase
ROS: Reactive oxygen species
miR-7: MicroRNA-7
MDSCs: Myeloid-Derived Suppressor Cells
GC: Gastric cancer
GERD: Gastroesophageal reflux disease
UCB: Urothelial carcinoma of the bladder
